# Aspergillosis and the role of mucins in cystic fibrosis

**DOI:** 10.1002/ppul.23618

**Published:** 2016-11-21

**Authors:** Abigail C. Cowley, David J. Thornton, David W. Denning, Alexander Horsley

**Affiliations:** ^1^ Wellcome Trust Centre for Cell‐Matrix ResearchUniversity of ManchesterManchesterUnited Kingdom; ^2^ Faculty of Biology, Medicine and Health, Division of Infection, Immunity and Respiratory Medicine, School of Biological SciencesUniversity of ManchesterManchesterUnited Kingdom; ^3^ Manchester Academic Health Science CentreThe University of ManchesterManchesterUnited Kingdom; ^4^ Manchester Adult CF CentreManchesterUnited Kingdom

**Keywords:** *Aspergillus fumigatus*, cystic fibrosis, mucus, mucins

## Abstract

The prevalence of aspergillosis in CF patients has until recently been underestimated, but increasing evidence suggests that it may play an important role in the progression of CF lung disease. In healthy airways, *Aspergillus fumigatus* can be efficiently removed from the lung by mechanisms such as mucociliary clearance and cough. However, these mechanisms are defective in CF, allowing pathogens such as *A. fumigatus* to germinate and establish chronic infections within the airways. The precise means by which *A. fumigatus* contributes to CF lung disease remain largely unclear. As the first point of contact within the lung, and an important component of the innate immune system, it is likely that the mucus barrier plays an important role in this process. Study of the functional interplay between this vital protective barrier, and in particular its principal structural components, the polymeric gel‐forming mucins, and CF pathogens such as *A. fumigatus*, is at an early stage. *A. fumigatus* protease activity has been shown to upregulate mucus production by inducing mucin mRNA and protein expression, and *A. fumigatus* proteases and glycosidases are able to degrade mucins. This may allow *A. fumigatus* to alter mucus barrier properties to promote fungal colonization of the airways and/or utilize mucins as a nutrient source. Moreover, conidial surface lectin binding to mucin glycans is a key aspect of clearance of *Aspergillus* from the lung in health but may be an important aspect of colonization, where mucociliary clearance is compromised, as in the CF lung. Here we discuss the nature of the mucus barrier and its mucin components in CF, and how they may be implicated in *A. fumigatus* infection. **Pediatr Pulmonol 2017;52:548–555.** © 2016 The Authors. Pediatric Pulmonology. Published by Wiley Periodicals, Inc.

## INTRODUCTION

Cystic fibrosis (CF) lung disease is characterized by chronic progressive airway infection, and clinically by the accumulation of airway secretions. At the level of the small airways, the combination of airway wall inflammation and thick luminal secretions results in airway blockage and remodeling. The role of bacterial pathogens such as *Pseudomonas aeruginosa* in this process has been the subject of extensive research. Only more recently, however has the impact of fungal organisms such as *Aspergillus* on CF airways been examined.^1^, ^2^ *Aspergillus*, in particular *Aspergillus fumigatus*, is one of the main fungal species found in CF airways. This filamentous fungus may act both as an allergen and an opportunistic pathogen in patients with CF, and can be isolated from airway secretions of up to ∼60% of CF patients.^3^, ^4^, ^5^ Chronic infection with *A. fumigatus* is an independent risk factor for hospital admissions, and sensitization to *A. fumigatus* has been linked to a greater decline in lung function and increased pulmonary exacerbations.^6^, ^7^, ^8^ However, due to a lack of sensitive culture methods it is likely that the prevalence of *A. fumigatus* in CF sputum has been underestimated.^9^

Part of the difficulty in defining the role of *A. fumigatus* in the progression of CF lung disease lies within the wide range of host responses that result in differing clinical states.^9^ Historically, *A. fumigatus* was regarded either as a colonizer of the airways or the cause of an exaggerated host allergic response, causing allergic bronchopulmonary aspergillosis (ABPA). A recent classification, based on a more detailed assessment of host (IgG, IgE) and airway infection (*Aspergillus* PCR and galactomannan antigen) markers, has provided new insights into the various responses of CF patients to *A. fumigatus* infection.^9^, ^10^ However, these data provide no insight into the long term clinical consequences of these different disease categories, nor about whether or how patients may switch between phenotypes, manifest more than one of these synchronously or resolve a disease state spontaneously. Current treatment for aspergillosis involves the use of antifungal agents such as azoles. However, these are all potentially toxic treatments, with a variable record of therapeutic success.^11^ Better understanding of the pathophysiology of host‐fungal interactions, particularly in the early colonization steps, may help in the development of new therapeutic strategies.

Airway colonisation by *A. fumigatus* occurs following inhalation of airborne conidia, asexual spores that are ubiquitous within the environment. These spores are frequently inhaled by humans and their small diameter (2 and 3µm) allows them to easily penetrate the lower airways of the respiratory tract.^12^ In healthy individuals, inhalation of *A. fumigatus* conidia is generally harmless, as they are efficiently removed from the airways by entrapment within airway mucus, phagocytosis by airway macrophages and removal by mucociliary clearance.^13^ Prior to swelling and then germination, they evoke no immune response.^14^ However, under circumstances where these innate defence mechanisms are compromised, as in CF, conidia persist within the airways. Here they may germinate and form hyphae that can invade the epithelial layer causing tissue damage.^12^, ^13^, ^15^ *A. fumigatus* secretes the complex carbohydrate galactosaminogalactan over its hyphal surface, which conceals β‐glucan from the immune system and mediates adherence to the galectin‐3 protein on the epithelial surface.^16^ Swollen conidia and hyphae also secrete allergens and gliotoxin, which have been shown to interfere with phagocytosis of inhaled conidia, preventing their elimination from the airways.^17^ Gliotoxin has been shown to slow ciliary beating in association with epithelial damage, perhaps through induction of apoptosis,^18^ with the consequence of reducing clearance of germinating conidia.

In order to colonize the airways effectively, conidia must ultimately adhere to the epithelium or basement membrane of the airway wall, interacting with extracellular matrix components such as laminin and fibrinogen.^19^ As the first point of contact for *A. fumigatus* following inhalation however, the mucus barrier is an important site of conidia‐host interactions. Details of these interactions between mucus and *A. fumigatus*, and how they change in the altered environment of the CF lung remain unclear, but may be key to understanding the susceptibility of CF patients to chronic *A. fumigatus* infection.

### The Mucus Barrier in Cystic Fibrosis

Mucus is a multifaceted secretion composed of water, inorganic salts, proteins, glycoproteins, and nucleic acids, and is a key component of the innate defence system.^20^ In healthy airways, this dynamic barrier traps inhaled pathogens and other foreign particulates, allowing them to be cleared from the airways by mucociliary transport and/or cough. In CF, the mucus barrier becomes static and adherent, providing an ideal environment for airborne pathogens (viral, bacterial, and fungal) and allowing them to flourish within the airways.^21^

Exactly how mucus abnormalities contribute to CF lung disease remains to be fully elucidated, as there are many different factors that must be considered. The defective gene in CF encodes for a cyclic adenosine monophosphate regulated chloride channel (CFTR) expressed on the apical surface of lung and other epithelial tissues. CFTR dysfunction creates an ionic imbalance across the airway epithelial layer that affects the hydration, pH and electrolyte and mucus concentration of the airway surface liquid (ASL); all of these factors have been shown to alter mucus properties and negatively impact on mucociliary transport.^21^, ^22^, ^23^, ^24^ Dehydration of the ASL and the resultant increased mucus concentration affects mucociliary clearance by “osmotically compressing” the periciliary layer and collapsing the cilia, leading to mucus stasis and airway obstruction.^21^, ^22^ Data generated from a pig model of CF has shown that decreased pH of the ASL (due to defective bicarbonate secretion through CFTR) and increased calcium concentration negatively impacted on mucociliary transport; decreased pH also directly inhibited the antimicrobial properties of the ASL.^24^ Moreover, this model has also shown that impaired mucociliary transport can be caused by abnormal tethering of mucus strands to the submucosal glands, with their failure to detach in CF preventing clearance from the airway surface.^23^

The properties of mucus are largely dependent on polymeric, gel‐forming mucins, which are the major macromolecular constituents of the barrier.^25^ In healthy airway mucus, MUC5AC and MUC5B are the major gel‐forming mucins, and are secreted mainly by goblet cells and the submucosal glands, respectively (MUC5B is also produced by goblet cells, although in smaller amounts).^26^, ^27^ Once secreted, these large O‐linked glycoproteins are responsible for the structural architecture and biophysical properties of mucus. In solution, mucins interact both with one another and with other molecules such as calcium, which plays an important role in mucin cross‐linking.^28^, ^29^ Sequence variations in the central repetitive exon of the *MUC5AC* gene have been associated with severity of CF lung disease,^30^ perhaps resulting in MUC5AC polymers that have increased capacity to form cross‐links that will influence mucus function in terms of barrier porosity, viscosity, and transport by cilia.^31^

Mucin polymers are adorned with an array of sugar side chains comprising monosaccharides including N‐acetylgalactosamine (GalNAc), N‐acetylglucosamine (GlcNAc), fucose, sialic acid, and galactose, which can be further modified by sulfation. Charge repulsion between the anionic groups on sulfated galactose and sialic acid residues causes mucins to adopt an expanded conformation, which is central to mucus gel formation and properties.^25^, ^32^ These glycans also provide a variety of carbohydrate epitopes that are able to bind pathogens and sequester them within the mucus barrier, as well as providing a protective shield against proteases, helping mucins to resist proteolytic degradation.^33^

Given the importance of airway secretions in the pathophysiology of CF, it is perhaps not surprising that changes in mucin properties have been observed in CF sputum (for a detailed overview see ref^34^). In brief, mucins isolated from CF sputum are on average smaller than those from healthy individuals, most likely due to their degradation by the many proteases present in the CF lung. Mucin glycosylation changes have also been observed, with some reports suggesting that a less acidic form of MUC5B predominates in CF sputum,^26^, ^35^ while others have reported increased acidity for some mucins in CF.^36^, ^37^

Although mucins are the main constituent of healthy mucus, the extent to which they contribute to the biophysical properties of CF sputum has been subject to some debate. Some studies have identified DNA as the main macromolecular constituent of CF sputum, and suggested that it may play a more significant role in defining its biophysical properties.^38^, ^39^ Moreover, it has been reported that mucin levels in CF sputum are decreased compared with those of healthy individuals.^40^ However, these early reports were based on sputum from patients chronically infected with *P. aeruginosa*. Sputum from patients with no history of *P. aeruginosa* infection appears to contain mucin levels similar to those of healthy individuals.^38^ Thus, the apparent low level of mucins measured in infected patients (using antibody based assays) is most likely due to the action of proteases secreted by *P. aeruginosa* (as well as other airborne pathogens), which degrade mucins^38^ and reduce their detection by mucin‐specific antibodies. More recently, using size exclusion chromatography and differential refractometry, mucin concentrations have been shown to be higher in CF secretions as compared with healthy secretions.^21^ Mass spectrometry also revealed that CF mucins become degraded at antibody recognition sites. This highlights the inaccuracy of immunological techniques in measuring mucin concentration in CF secretions, and may explain the seemingly low levels of mucins measured in other studies.^21^ Indeed, quantitative mass spectrometry investigation of the secretome from CF bronchial epithelial cells has shown an increase in both MUC5AC and MUC5B expression and secretion, even in the absence of inflammation and infection, suggesting that the CF defect may be directly related to increased mucin production.^41^ In light of these findings, the biophysical properties of CF sputum may be more reliant on the state of mucins than previously reported. Although the precise impact of, and interaction between the different macromolecular components of sputum is not fully understood, it is becoming increasingly clear that mucins are an important constituent of CF airway secretions, both in early and advanced disease.

### Interactions Between *Aspergillus* and Airway Mucins

**Figure 1:**
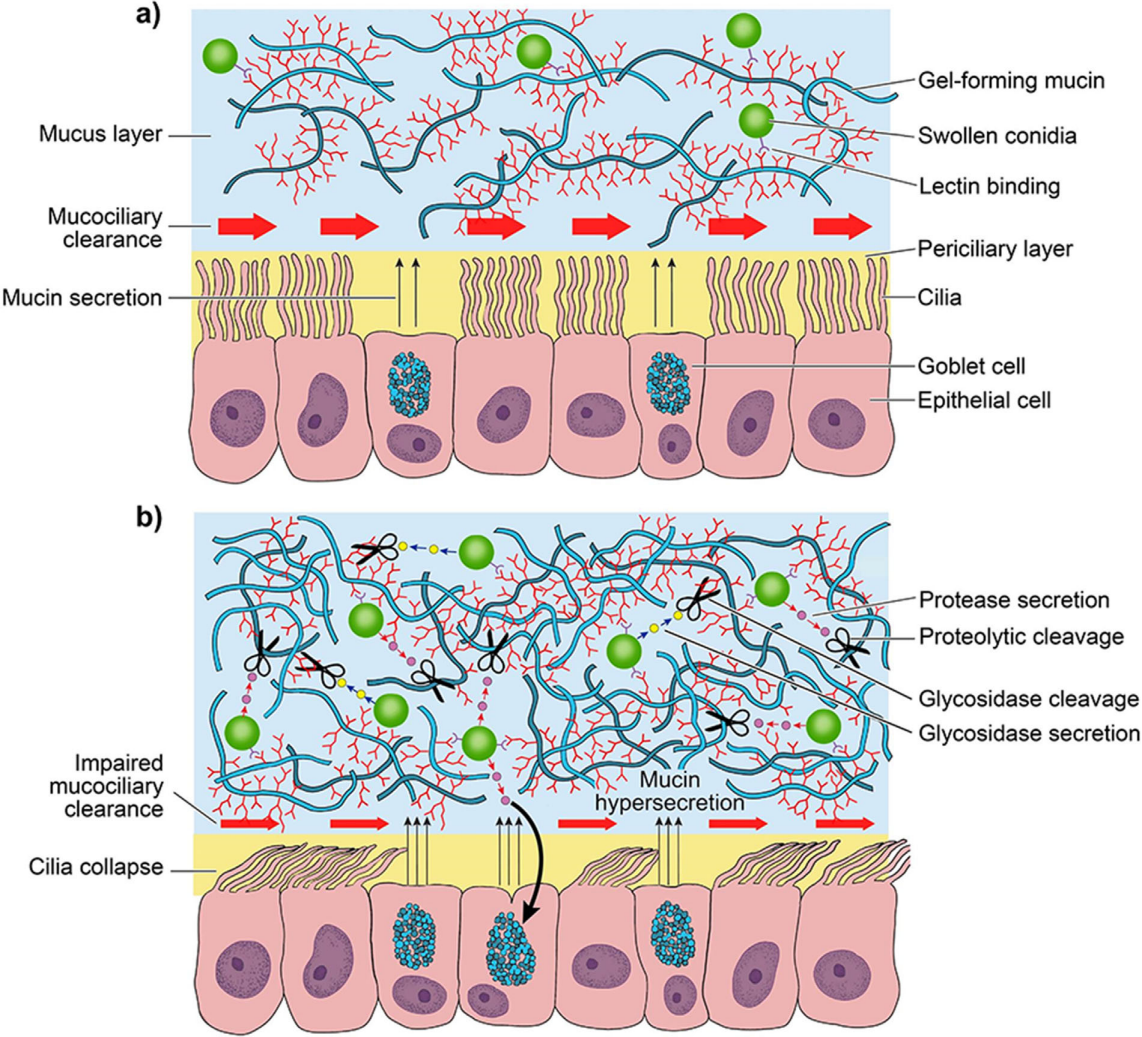
a) Inhaled conidia are efficiently removed from healthy airways by mechanisms such as mucociliary clearance. This is perhaps facilitated by binding of conidial lectins (e.g. FleA) to glycan moieties on gel‐forming mucins (MUC5AC and MUC5B), allowing them to become trapped within the mucus barrier and subsequently cleared from the airways.^50^ b) In cystic fibrosis mucociliary transport is impaired, preventing the elimination of inhaled pathogens from the airways. Here conidia may secrete proteases and glycosidases that degrade mucin protein and carbohydrate, in order to compromise the protective properties of the mucus barrier. *A. fumigatus* protease activity has also been shown to induce MUC5AC expression in airway epithelial cells, which may represent a mechanism of host defense against *A. fumigatus*.

The alterations in mucus properties and ciliary function in CF airways result in reduced mucus clearance, and this static barrier permits colonization of the airways by inhaled pathogens such as *A. fumigatus*.^42^ Initial adhesion of *A. fumigatus* within the airways involves the binding of specific sugars or lectins on the conidial surface^19^, ^43^, ^44^ (Fig. 1A). The precise mechanistic details of this process are largely unknown, though early studies suggested a role for negatively charged carbohydrates (particularly sialic acid) on the conidial surface.^43^, ^44^ More recent work has reported that galactosaminogalactan acts as a fungal adhesin in *A. fumigatus*, and this polysaccharide has also been shown to suppress host inflammatory responses.^16^ Tronchin et al. identified a sialic acid‐specific lectin in *A. fumigatus* conidia, which may also be involved in conidial adhesion to the airway mucosa via components such as laminin and fibrinogen.^45^ Mucins themselves are likely targets for conidial binding (Fig. 1A), as their glycan chains are often terminated in sialic acid residues. Indeed, sialic acid‐dependent interactions with mucins have been observed in other pathogens such as *P. aeruginosa*,^46^ and may represent a common mechanism of pathogen binding prior to colonization of the airways. Subsequent chronic infections are largely associated with the formation of surface‐attached biofilms, though a role for non‐attached aggregates in such infections has also been proposed.^47^ *A. fumigatus* may form fungal balls, otherwise know as aspergillomas during colonization of epithelial surfaces such as sinus mucosa or inside the lung cavity. Admixed with fungal hyphae are extensive biofilm materials including glycocalyx, galactomannan, melanin, and extracellular DNA, as well as mucus and cellular debris.^48^ It is likely but not well substantiated yet that *A. fumigatus* can form a mixed biofilm with bacteria in the lower airways.^49^ The role of this potential interaction in facilitating Aspergillus infection is not well established, but it is probable that it also takes place in CF airways.

Conidial surface lectins also play key roles in pathogenesis. For example, *Aspergillus* has been proposed to express a fucose‐specific lectin (FleA, or AFL) on the conidial surface.^50^, ^51^ FleA has recently been shown to bind fucosylated glycans on airway mucins, and also appears to be required for binding and phagocytosis of conidia by alveolar macrophages. In a mouse model of *A. fumigatus* pneumonia, treatment with Δ*fleA* conidia led to increased lung injury and infection, highlighting a role for FleA as a pathogen‐associated molecular pattern that is important for host defence against *A. fumigatus* infection.^51^ Thus, binding of inhaled pathogens to mucins may normally act as a protective mechanism, ensuring they are trapped within the mucus layer and unable to reach the underlying epithelium, before being removed by mucociliary clearance (Fig. 1A). In healthy airways, these interactions may be transient and insufficient to allow *A. fumigatus* to germinate. However in CF, persistence of *A. fumigatus* within the airways may lead to germination of conidia and the release of virulence factors (Fig. 1B), making the development of a host immune response more likely. An in vitro study of Aspergillus–epithelial interactions found that CF epithelial cells exhibit defective uptake and killing of *A. fumigatus* conidia, and excessive conidia‐induced apoptosis.^52^ Thus in the absence of effective mucociliary clearance, *A. fumigatus* may have a significant impact upon airway epithelial integrity.

### Impact of *Aspergillus* on Airway Mucins

As well as forming interactions with mucus barrier components, *A. fumigatus* may also manipulate mucins in order to establish a niche within the airway mucosa. *Aspergillus* species produce a variety of proteases which are found either intracellularly, tethered to the cell wall or secreted.^53^ A number of these have been identified as putative virulence factors, and appear to play a role in the development of allergic diseases such as ABPA.^17^, ^54^ Proteolytic degradation of mucins has previously been observed in *Candida albicans* and *P. aeruginosa*,^55^, ^56^ and *A. fumigatus* has also been shown to degrade both mucin protein and carbohydrate.^57^ However, this has only been shown using commercially available mucins, which exhibit lower molecular weights and contain more impurities than intact mucins isolated under controlled conditions from “fresh” mucus secretions.^58^ The action of *Aspergillus* proteases on intact airway mucins has not yet been explored, but there are a number of reasons why *A. fumigatus* may benefit from degrading mucin proteins. One possibility is the utilization of mucins as a carbon source, which has been shown to occur in a number of bacteria.^59^ Degradation of mucins also alters the biophysical properties of mucus, perhaps to provide a more favorable niche, allowing *A. fumigatus* to penetrate and colonize the mucus barrier more easily. As well as utilizing proteases to degrade mucins, *A. fumigatus* also secretes glycosidases that degrade mucin glycans^57^ (Fig. 1B). Degrading mucins in this way could allow *A. fumigatus* to utilize mucin glycans as a nutrient source, and act as a mechanism for inhibiting entrapment within the airway mucus barrier in order to prevent elimination from the airways. In the rich microbiome of the CF airway the impact of individual proteases and glycosidases cannot be considered in isolation, and infection with *A. fumigatus* may depend on the action of bacterial pathogens within the lung to compromise airway defenses, or vice versa. Antibiotic treatment of bacterial infections has been shown to cause a significant decrease in the level of *Aspergillus* in CF sputum, despite having no direct antifungal action.^60^ However, *P. aeruginosa*, particularly CF isolates, has been shown to inhibit *A. fumigatus* biofilm formation.^61^ Thus, elimination of bacterial infections may in some cases worsen the fungal burden within the CF lung, highlighting the complex nature of the interaction between host, bacteria, and fungi in the lower airways in CF.

Another mechanism by which *A. fumigatus* proteases can alter mucus barrier properties is by altering mucin gene expression. Using DNA microarray analysis, Oguma and coworkers showed that the serine protease activity of *A. fumigatus* is able to induce MUC5AC mRNA and protein expression in airway epithelial cells, via the sequential activation of TNF‐α‐converting enzyme (TACE), TGF‐α, and epidermal growth factor receptor (EGFR).^54^ In line with this, chronic exposure to *A. fumigatus* has been shown to induce MUC5AC mRNA and protein expression in asthmatic rats.^62^ Upregulation of MUC5AC in response to *A. fumigatus* infection may thus be an important host defence mechanism. In support of this contention, upregulation of MUC5AC has been seen in other host–pathogen interactions. For instance, de novo expression of intestinal Muc5ac in mice occurs in response to acute infection with the murine parasitic nematode *Trichuris muris*, and this response is critical for the prevention of chronic infection.^63^ This may be in part due to alterations in the structural organization of mucus, as Muc5ac‐deficient mice possess a more porous mucus barrier.^63^ Similarly, upregulation of MUC5AC in the human lung during *A. fumigatus* infection may represent a mechanism of host defence.

To date, *Aspergillus*‐related research has focused mainly on MUC5AC, rather than the other major respiratory mucin MUC5B. Studies have shown that Muc5b is required for airway defence in mice, playing a crucial role in mucociliary clearance and antibacterial responses.^64^ Thus, it is likely that MUC5B plays a similar role in human airways, and is important for host defence against *A. fumigatus*. Future work must explore this relationship further, investigating the effects of *A. fumigatus* on MUC5B and vice versa.

## SUMMARY

Airway mucus, and its constituent mucins, comprises the first line of airway defence, and the first component encountered by inhaled fungal spores. Although *A. fumigatus* and the airway mucus barrier appear to be able to interact and influence each other, many questions remain surrounding the role of this relationship in CF progression. Does *A. fumigatus* bind directly to mucins? If so, do these interactions promote airway colonisation or do mucins sequester *A. fumigatus* to hinder its pathological effects? Furthermore, does *A. fumigatus* degrade components of the mucus barrier as a source of nutrients, or in order to further compromise its protective properties? As *A. fumigatus* does not colonize CF lungs in isolation, it is also important to consider the interplay that exists between *A. fumigatus* and other major pathogens such as P. aeruginosa, and how this may also determine its effects on CF airways. Studying these processes in detail will provide us with a valuable understanding of aspergillosis in CF, and may ultimately aid the development of novel therapeutic agents to target such infections and improve patient health.
